# A Novel 3D Probe for Near-Field Scanning Microwave Microscopy

**DOI:** 10.3390/s26030995

**Published:** 2026-02-03

**Authors:** Ali M. Almuhlafi, Omar M. Ramahi

**Affiliations:** 1 Department of Electrical Engineering, College of Engineering, King Saud University, Riyadh 12372, Saudi Arabia; aalbishi@ksu.edu.sa; 2 Department of Electrical and Computer Engineering, University of Waterloo, Waterloo, ON N2L 3G1, Canada

**Keywords:** complementary split-ring resonators, field-spread function, near-field scanning microwave microscopy, point-spread function

## Abstract

Near-field scanning microwave microscopy (NSMM) offers the ability to probe local electromagnetic properties beyond the classical Abbe diffraction limit, but achieving high resolution over practical scan areas remains challenging. In this work, we introduce a novel three-dimensional (3D) NSMM probe consisting of a split-ring resonator (SRR) coupled to a microstrip line and loaded with vertically extended metallic bars. The 3D loading enhances electric-field localization in the sensing region by introducing field singularities. Full-wave numerical simulations are used to extract the field-spread function (FSF) of the probe and to quantify how probe geometry, stand-off distance, and bar dimensions control the FSF and its spatial-frequency (k-space) content. An imaging model is then developed in which the NSMM image is represented as a convolution between the object and FSF in one and two dimensions. This framework demonstrates that progressively localized FSFs, obtained through 3D loading and resonator miniaturization, systematically improve image fidelity and preserve higher spatial frequencies. The probe is fabricated using printed circuit board technology (PCB) with vertically attached metallic bars, and its performance is validated by imaging a dielectric slab containing a cylindrical air-filled void. The measured line profiles and two-dimensional images are in good agreement in general characteristics with the convolution-based model, confirming that the proposed 3D SRR-based probe operates as a spatial filter whose engineered near-field distribution governs the achievable resolution in NSMM imaging.

## 1. Introduction

Composite materials are increasingly used in advanced engineering systems, particularly in aerospace platforms, high-speed aircraft, and missile radomes, due to their excellent mechanical stability, low dielectric loss, and thermal robustness under extreme operating conditions [1,2]. Radome structures in supersonic and hypersonic vehicles are routinely exposed to severe acceleration, aerodynamic drag, and environmental degradation, including erosion, moisture, and thermal loads exceeding 350 °C [2]. These factors can introduce spatially varying dielectric properties that directly affect the transmission of electromagnetic waves, the performance of antennas, and the overall electromagnetic integrity of such systems.

Beyond radome applications, spatially engineered permittivity composite materials have been proposed for electrically thin flat lenses and reflectors [3,4]. The development of advanced additive manufacturing and 3D printing technologies has accelerated the fabrication of heterogeneous dielectric structures [5,6]. However, ensuring electromagnetic performance requires accurate knowledge of the local permittivity distribution of the material. Small deviations in dielectric properties can dramatically affect the behavior of lenses, reflectors, and other EM components that rely on controlled spatial gradients [3,4]. Thus, the ability to scan, image, and quantify local electromagnetic properties, particularly in composite materials with irregularities or defects, remains a key area of active research.

The observation of subwavelength spatial variations in composite dielectric materials presents a significant challenge, which can be theoretically quantified using the classical Abbe diffraction limit introduced by Ernst Abbe in 1873 [7,8]. Abbe demonstrated that, under ideal and aberration-free conditions, the resolution of a light microscope imaging a periodic grating is constrained by the illumination wavelength and the numerical aperture (NA) of the objective lens, yielding the well-known expression

(1) $$d=\frac{\lambda }{2\;\mathrm{NA}}$$

For a microscope with NA≈1, the minimum resolvable separation reduces to d≈λ/2. He also noted that resolution can be improved, for example, through the use of shorter-wavelength illumination.

In 1928, E. H. Synge proposed a method to reach ultramicroscopic resolution, marking one of the earliest attempts to surpass the Abbe diffraction limit [9]. Building on Synge’s concept, Ash and Nicholls later demonstrated experimentally, at microwave frequencies, that two objects can be resolved with a spatial resolution well beyond the classical Abbe limit by scanning a 1.5 mm diameter aperture at 10 GHz. These seminal contributions laid the foundation for modern near-field scanning probe microscopies [10].

A wide set of microscopy techniques have been applied to surface and subsurface characterization, including scanning electron microscopy (SEM) [11], atomic force microscopy (AFM) [12,13,14], scanning tunneling microscopy (STM) [15,16,17], near-field scanning optical microscopy (NSOM) [18,19,20], scanning capacitance microscopy (SCM) [21,22], and near-field immersion microwave microscopy (NFIMM) [23]. Although powerful, most of these methods either lack penetration depth, require conductive samples, or operate outside the microwave regime. Near-field scanning microwave microscopy (NSMM) addresses many of these limitations by enabling detection and imaging of subsurface features due to microwave penetration [24], quantitative dielectric characterization, operation on conductive and non-conductive materials, and non-destructive scanning of engineered composites. Several NSMM architectures have been reported [25,26,27,28,29,30,31,32,33,34,35,36,37,38], each with unique strengths but also notable limitations.

Open-ended coaxial probes infer material properties from the reflection coefficient using equivalent-circuit models. Their spatial resolution is typically in the millimeter-to-centimeter range, governed by the aperture diameter rather than the operating wavelength [39]. Although these probes bypass the diffraction limit, their resolution remains fundamentally constrained by the physical tip size. Imtiaz et al. describe a classical NSMM system using an open-ended coaxial resonator tip of effective radius *D*, a vector network analyzer (VNA) and a decoupling capacitor [25]. Its resolution lies between the free-space wavelength λ and *D*, requiring extremely small stand-off distances *r* (with D≤r≪λ). Achieving such distances requires complex feedback mechanisms (based on STM or AFM) and careful calibration [25].

Alternatively, microstrip-line resonators with small tips have been proposed as probes, achieving resolutions on the order of 0.4 μm [33]. Their advantages include flexible substrate selection and controlled coupling [33,40]. However, these probes face challenges, including the need for a highly stable microwave source and the difficulty of interpreting measurement results, since the measured signal depends on the tip–sample interaction and may not directly correspond to the material properties of interest. In addition, the technique requires careful calibration and optimization to ensure reliable and accurate measurements [33].

NSMM systems based on open-ended waveguides with slots have also been investigated [41,42,43,44]. In these scanners, spatial resolution is fundamentally limited by slot aperture dimensions and is typically greater than 100 μm [39], making subwavelength resolution challenging unless significantly higher operating frequencies are used [45]. These systems generally rely on voltage standing-wave measurements detected by diode detectors [41], but measured signals can be affected by noise, air gaps, and other uncertainties. In addition, open-ended waveguide probes are sensitive to the orientation of the sample under test (SUT) [41,42], which may distort the measured response. While all of the aforementioned techniques have been used successfully for material scanning and imaging, they share a common limitation: achieving large-area, high-resolution scanning remains challenging [39].

Electrically small resonators, such as split-ring resonators (SRRs), have also been proposed as an alternative solution for material scanning, sensing, and imaging, particularly when large-area scanning is impractical [46,47]. One approach incorporates multiple SRRs, where selected resonators are used for scanning while others function as reference elements to assist with calibration and drift compensation [46,47]. Wiwatcharagoses et al. introduced multiple resonators for dielectric imaging, although the technique was not explored further [46]. Building on this work, Mukherjee et al. proposed a similar configuration with extended SRR splits and claimed that edge-coupling-based interrogation improves resolution since the sensing region becomes determined by the split gap rather than the top surface of the resonator [47]. However, edge-coupling-based scanning had already been explored in [46], and despite the extended splits, the resonators remained strictly planar.

As established in [48,49,50], planar SRRs confine the majority of their electric field within the dielectric substrate, limiting sensitivity when the sample does not occupy the dominant field region. In [50], the SRR splits were extended vertically relative to the resonator plane, enhancing the electric field in the sensing region and significantly improving sensitivity. Additionally, elevating the sensing region eliminated unwanted interaction with the excitation transmission line, thereby reducing parasitic loading effects. Similar loading effects were investigated in [51].

A two-port SRR-based measurement system was described in [39], where SRRs were excited using two magnetic loop probes. Although the study provided a schematic of the scanning probe, it did not specify how the distance between the loop probes and the SRRs was maintained during scanning, despite this spacing directly influencing both the coupling coefficient and resonance frequency. Furthermore, because the scanning probe was required to physically contact the sample surface, continuous scanning becomes difficult and potentially impractical. The study did not present images of the fabricated probe, leaving implementation details unclear. Additionally, discrepancies between experimental and theoretical results were reported for one of the resonators, attributed to uncertainties introduced by the SRR–sample contact procedure [39].

In this work, we propose a novel three-dimensional (3D) probe for near-field scanning microwave microscopy (NSMM), consisting of a microstrip line coupled to an SRR loaded with two vertically extended metallic bars. This design adopts the 3D loading concept previously introduced for fluidic sensing applications [50]. The proposed architecture offers several advantages:

1. Enhanced electric-field localization: The vertically extended metallic bars intensify the electric field in the sensing region, improving sensitivity.
2. Scalability for large-area scanning: The microstrip-line excitation enables multiple resonators with distinct resonance frequencies to be integrated along a single line.
3. Elimination of feedline–sample interaction: Elevating the sensing region minimizes parasitic coupling to the transmission line.
4. Frequency tunability and miniaturization: The bars act as loading capacitors that reduce the resonance frequency, facilitate sensor miniaturization, and suppress unwanted radiation.
5. Electric-field singularity at sharp bar tips: The sharp metallic edges induce electric-field singularities, yielding spatial resolution governed by the local field distribution rather than the physical probe dimensions.

To evaluate the probe performance, we conducted full-wave simulations in HFSS-Ansys [52], analyzing the spatial electric-field distributions in the sensing region. For the first time in this context, a Fourier transform (e.g., for spatial-frequency decomposition) was employed to quantify the high-*k* spatial components that dictate achievable resolution. The probe was fabricated using printed circuit board (PCB) technology, with the SRR loaded by vertically attached aluminum bars bonded using conductive adhesive. The fabricated probe was used to image a dielectric slab containing a void-type defect, demonstrating its effectiveness as an NSMM probe.

## 2. Near-Field Scanning Microwave Microscopy: Theory, Design, and Synthesis

Electrically small resonators, including split-ring resonators (SRRs) and complementary SRRs (CSRRs), are widely used in metamaterials and near-field sensing applications [50,53,54,55,56,57,58,59,60,61]. At resonance, the electromagnetic fields are concentrated in the near vicinity of these structures [53], enabling their use in sensitive detection schemes.

**Figure 1:**
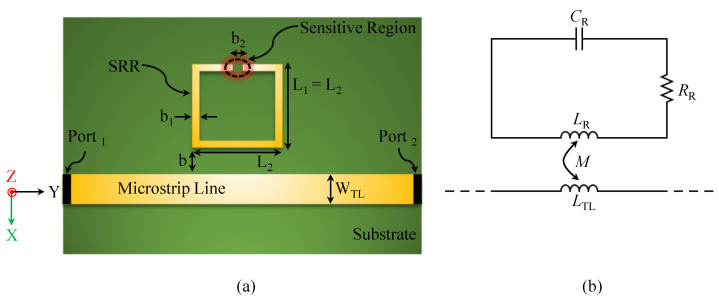
The SRR-based sensor. (**a**) The schematic top view of a two-port microstrip line loaded with SRR. (**b**) The circuit model of the system at the resonance frequency.

SRRs can be excited by the quasi-TEM mode of a two-port microstrip transmission line (TL). The resonator, consisting of a ring with a split, is placed adjacent to TL and is excited primarily by the magnetic field normal to its plane. Figure 1a shows the top view of TL and SRR, where $L_{1}$ and $L_{2}$ are the side lengths of the resonator, b is the gap between TL and SRR, $W_{\mathrm{TL}}$ is the TL width, $b_{1}$ is the resonator-strip width, and $b_{2}$ is the width of the ring split (the region with the highest electric-field concentration). Figure 1b illustrates the equivalent-circuit model [62]. The resonance frequency is

(2) $$f_{z0}=\frac{1}{2\pi \sqrt{L_{R}C_{R}}},$$

where $L_{R}$, $C_{R}$, and $R_{R}$ represent the inductance, capacitance, and resistance of the resonator, respectively, and $L_{\mathrm{TL}}$ is the inductance per unit length of TL. Although approximate, this model provides insight into how resonance depends on geometry and the surrounding medium.

The capacitance $C_{R}$ of planar resonators includes contributions from the substrate and air [49]:

(3) $$C_{R}=\beta_{1}C_{\mathrm{sub}}+\beta_{2}C_{\mathrm{air}}$$

where $\beta_{1}$ and $\beta_{2}$ ($\beta_{1}+\beta_{2}=1$ or $\beta_{2}=1-\beta_{1}$) denote the contribution of each component. For sensing, it is desirable that $\beta_{2}\gg \beta_{1}$, since $C_{\mathrm{air}}$ is the portion that interacts with materials under test (MUTs). When $\beta_{2}$ approaches 1, $C_{R}\approx C_{\mathrm{air}}$, yielding maximum sensitivity. This concept underlies previously reported three-dimensional capacitor-based enhancements for CELC [49] and SRR [50] fluidic sensors.

**Figure 2:**
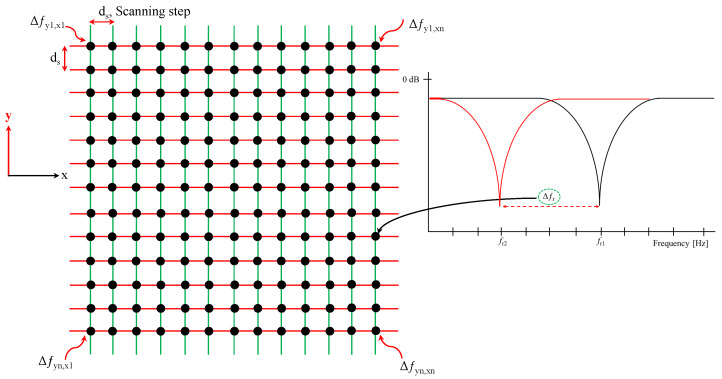
The general procedure to be followed for constructing 2D image of an object using resonance-frequency shift (Δf). The red and green lines are the virtual scanning lines in the x and y directions, respectively.

Here, a similar 3D capacitor-based approach is used to design an NSMM probe for surface detection and, ultimately, imaging. The sensing mechanism is based on measuring the resonance-frequency shifts (Δf) relative to a reference state. Mapping Δf over the *x*-*y* plane yields a two-dimensional image, as illustrated in Figure 2.

**Figure 3:**
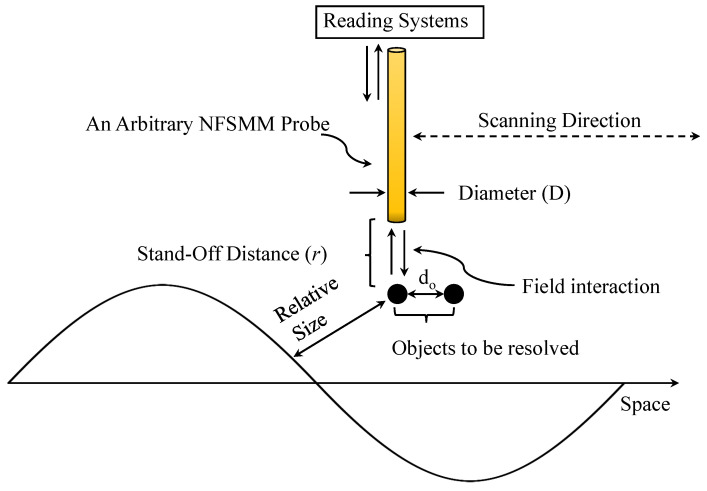
The illustration of an arbitrary NSMM probe that is used to resolve two separated objects by $d_{0}$.

As briefly discussed before, in NSMM systems, spatial resolution is determined by the probe size (diameter *D*) and stand-off distance *r*, not by the operating wavelength [25]. Figure 3 illustrates the following concept: two objects separated by $d_{o}$ are resolvable even when $d_{o}<\lambda /2$ (the Abbe limit [7]) provided the NSMM near-field criteria

**Figure 4:**
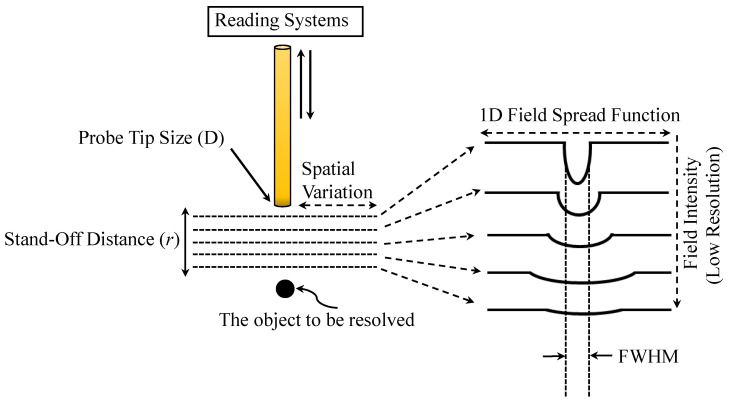
The relationship between the stand-off distance (*r*) and FSF of the spatial field variation (in 1D distribution).

(4) D≤r≪λ

are satisfied [25]. Thus, *D* and *r* impose the fundamental resolution limits. Figure 4 illustrates the relationship between the stand-off distance *r* and the 1D field-spread function (FSF). FSF generally represents the two-dimensional spatial distribution of the electric field in the sensing region. As *r* increases, FSF becomes wider, meaning that the full width at half maximum (FWHM) of the field distribution increases. Therefore, NSMM resolution is better expressed in terms of FSF’s FWHM rather than probe size. When FSF is engineered to have a small FWHM (possibly smaller than *D*), a higher resolution becomes achievable.

**Figure 5:**
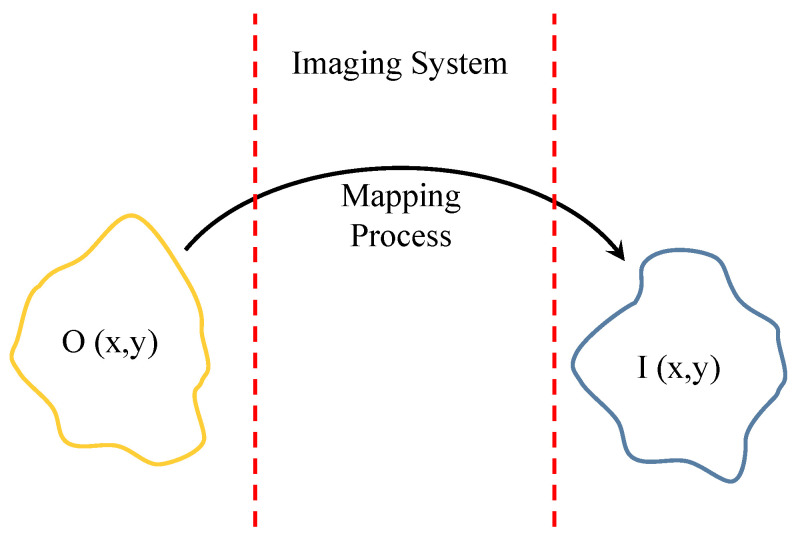
An imaging system utilized to project an object from object space to image space.

A smaller FWHM corresponds to a field distribution containing higher spatial-frequency components. This can be quantified by decomposing FSF using the Fourier transform:

(5) $$FSF\left(f_{x},f_{y}\right)=\mathrm{FT}\left\{FSF\left(x,y\right)\right\}$$

(6) $$FSF\left(f_{x},f_{y}\right)=\int_{-\infty }^{\infty }\int_{-\infty }^{\infty }FSF\left(x,y\right)\;e^{-i2\pi (f_{x}x+f_{y}y)}\;dx\;dy$$

where $f_{x}$ and $f_{y}$ are the spatial frequencies. This decomposition shows how much high-k content the probe supports, a property directly related to subwavelength resolution. Probes with strong high-frequency spectral components can more effectively resolve fine spatial features. Let us now represent an object in the object space using the function O(x,y) (the object’s spatial distribution in object space), whereas I(x,y) represents the object in the image space. The projection of the object into the image space is illustrated in Figure 5. If we assume the system is linear time-invariant (LTI), the system can be represented by the impulse response h(x,y). Hence, the mapping process can mathematically be represented by convolution as follows:

(7) I(x,y)=O(x,y)∗h(x,y)

Here, the field-spread function FSF(x,y) is defined as the normalized spatial distribution of the electric field in the sensing region produced by the probe at a given stand-off distance. The function O(x,y) represents the spatial distribution of the object’s electromagnetic properties in the object space.

However, if we assume the imaging system works as a linear spatial filter that is represented by FSF(x,y), then the image can be constructed as

(8) I(x,y)=O(x,y)∗FSF(x,y)

or

(9) $$\mathrm{Image}\left(x,y\right)=\int_{-\infty }^{\infty }\int_{-\infty }^{\infty }O\left(x-x^{\prime },\;y-y^{\prime }\right)\;FSF\left(x^{\prime },y^{\prime }\right)\;dx^{\prime }\;dy^{\prime },$$

The quality of the reconstructed image depends on FSF; for example, a Gaussian FSF produces a blurred image.

**Figure 6:**
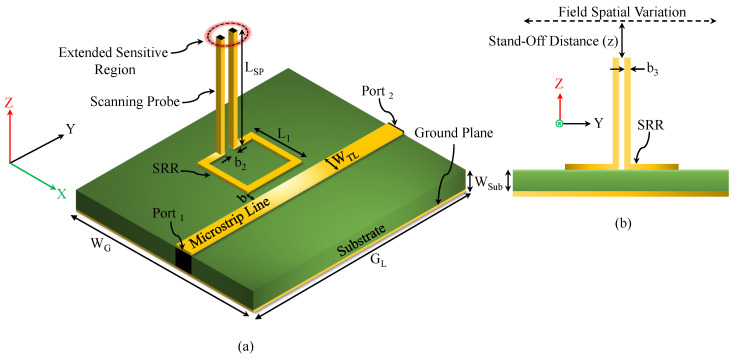
The SRR-based sensor. (**a**) The schematic top view of a two-port microstrip line loaded with SRR. (**b**) The side view.

**Figure 7:**
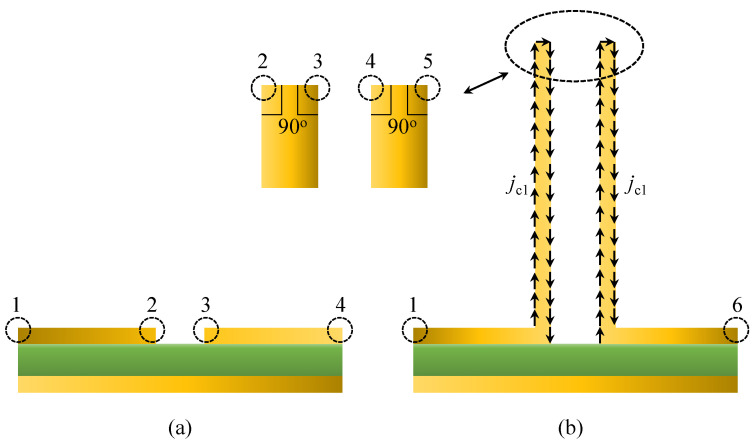
The schematic of the NSMM probe encounters six sharp conducting edges (denoted as 1, 2, 3, and 4, 5, and 6) (**a**) without vertically extended metallic bars (**b**) with, along with the expected surface current density.

Figure 6a,b show the two-port microstrip line coupled to an SRR and loaded with two metallic bars. As reported in [50], a strong electric field develops between the bars, forming a sensitive region suitable for scanning. The surface current density encounters six sharp conducting edges (denoted as 1, 2, and 3 and 4, 5, and 6) of angle 90° with and without vertically extended metallic bars, illustrated in Figure 7.

As a diffraction problem, the electric field becomes singular at these sharp edges [63]. Although the theoretical field magnitude can approach infinity, the Meixner edge condition ensures that the electromagnetic energy remains finite over any bounded region (e.g., integrable electromagnetic energy density over any bounded region). Because the strong field concentration is directly related to the geometry of the edges, modifying these edges allows engineering of FSF to obtain higher-resolution NSMM images.

## 3. Numerical Analysis

### 3.1. Field-Spread Function Analysis of 90° Sharp Edges

Generally speaking, the resonance frequency of a square planar SRR coupled to a microstrip line is strongly dependent on its side length. To ensure compatibility with the measurement capabilities of the available vector network analyzer (VNA) while maintaining a compact probe footprint, the resonator side length was selected as 7.5 mm, yielding a resonance below 10 GHz. Aside from that, there is no particular reason for choosing this length of 7.5 mm. A 50 Ω microstrip line was implemented for excitation, corresponding to a trace width of 1.63 mm on a Rogers RO4350 substrate ($\varepsilon_{r}=3.66$, tanδ=0.0031, thickness = 0.76 mm). The probe length $L_{\mathrm{SP}}$ was kept as a variable for further investigation. All relevant geometric parameters appearing in Figure 1 and Figure 6 are summarized in Table 1.

**Figure 8:**
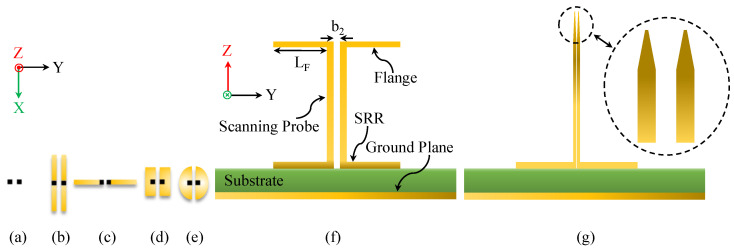
Different types of tips. (**a**) Square tip. (**b**) Rectangular flanges in the y-direction (attached to the square tip). (**c**) Rectangular flanges in the y-direction. (**d**) Flanges where the flanges are extended in the x- and y-directions. (**e**) Half-circle flange. (**f**) The scanning probe with rectangular flanges in the y-direction. (**g**) The tip of a needle-shaped extension.

One advantage of the proposed 3D-loaded SRR probe is its flexibility in accommodating different tip geometries depending on the application. As illustrated in Figure 8, the sensing tip can be modified by incorporating rectangular or circular flanges or even a needle-shaped extension. Each tip configuration produces a distinct FSF, which can be predicted through full-wave electromagnetic simulations prior to fabrication. This flexibility also allows the creation of a tunable scanning system in which the SRR structure remains fixed while interchangeable tips provide different FSFs for different imaging tasks.

**Figure 9:**
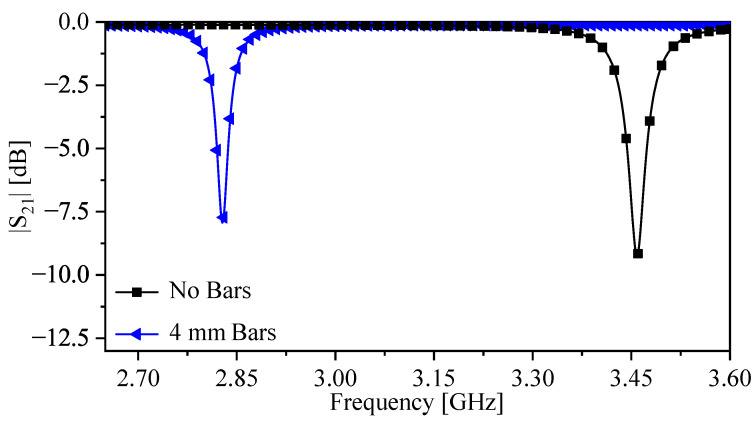
The response of the sensor with ($L_{\mathrm{SP}}$ = 4 mm) and without vertically extended metallic bars, that is presented in Table 1.

Figure 9 presents the numerical results of the observed transmission coefficient $|S_{21}|$ for the sensor with and without vertically extended metallic bars at $L_{\mathrm{SP}}=4$ mm. The resonance frequencies are 3.458 and 2.828 GHz, respectively, indicating an 18.218% reduction in resonance frequency due to the added bar capacitance.

Furthermore, unwanted environmental coupling is a well-known limitation in near-field scanning microwave microscopy (NSMM). Even with a controlled tip–sample spacing, the measured signal always includes contributions from the surrounding environment, which reduces accuracy [25]. To address this, our SRR-based probe is intentionally designed to minimize radiation. Although SRRs are naturally low-radiation structures, adding metallic bars further suppresses radiation and reduces environmental interaction. HFSS simulations show that the radiation efficiency drops from 0.95% (without bars) to 0.28% (with bars), confirming that the modified SRR couples much less to the environment. This helps the probe focus more on the true local response of the material under test.

**Figure 10:**
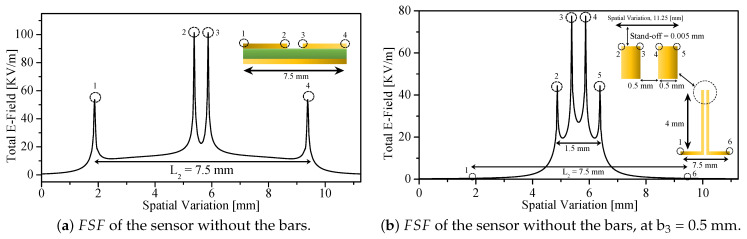
FSF as a function of the spatial variation, at $L_{2}$ = 7.5 mm and $b_{2}$ = 0.5 mm.

To analyze the influence of the sharp 90° edges on the field localization, the 1D FSF was extracted at a stand-off distance of 0.005 mm for both versions of the probe. Because the sensing region is symmetric, FSF was sampled along the midline (5.625 mm) over a span of 11.25 mm. Figure 10a (without the bars) and Figure 10b (with the bars) show the extracted FSFs.

In the sensor without bars (resonating at 3.458 GHz), four sharp edges, labeled points 1 through 4 in Figure 10a, give rise to pronounced electric-field singularities. Points 2 and 3 correspond to the two edges that form the SRR split, while points 1 and 4 correspond to the outer edges of the resonator side that contains the split. The total magnitude of the electric field at points 2 and 3 is 101.4 kV/m, while at points 1 and 4, it is 53.6645 kV/m. The absolute difference is therefore 47.774 kV/m. Because the imaging performance depends on the *relative* spatial variation of the field rather than its absolute magnitude, the relative difference referenced to the peak value is a more meaningful metric. This relative contrast equals 47.1%, indicating a substantial variation between the split-region singularity and the far-edge singularity.

In contrast, the sensor with vertically extended metallic bars (resonating at 2.828 GHz) exhibits six significant field points, labeled 1–6 in Figure 10b. Points 1, 3, 4, and 6 share the same horizontal spatial locations as points 1 and 4 in the unloaded SRR, while points 2 and 5 arise due to the presence of the bars and represent new field-localization sites. The vertical distance from the SRR plane to the sampling plane at the stand-off height is 4 mm + 0.005 mm = 4.005 mm. The absolute field values at these points are as follows:

- Points 1 and 6: 0.419 kV/m;
- Points 2 and 5: 44.38 kV/m;
- Points 3 and 4: 77.52 kV/m.

Thus, the absolute difference between the maximum and minimum field magnitudes is 77.101 kV/m. When referenced to the peak field (77.52 kV/m), the relative variation is 99.46%. This dramatic increase in contrast clearly demonstrates the technical advantage of vertically extended metallic bars: the field at the far edges (points 1 and 6) is suppressed by nearly two orders of magnitude relative to the concentrated sensing region (points 2 to 5), equivalently,

$$\log_{10}\left(\frac{{\left|E\right|}_{\mathrm{edge}\;}}{{\left|E\right|}_{\mathrm{center}\;}}\right)=\log_{10}\left(5.4\times 10^{-3}\right)\approx -2.27$$

As a result, FSF becomes significantly more localized around the central sensing region, reflecting the stronger electromagnetic confinement produced by the four 90° conducting edges at the tips of the bar.

**Figure 11:**
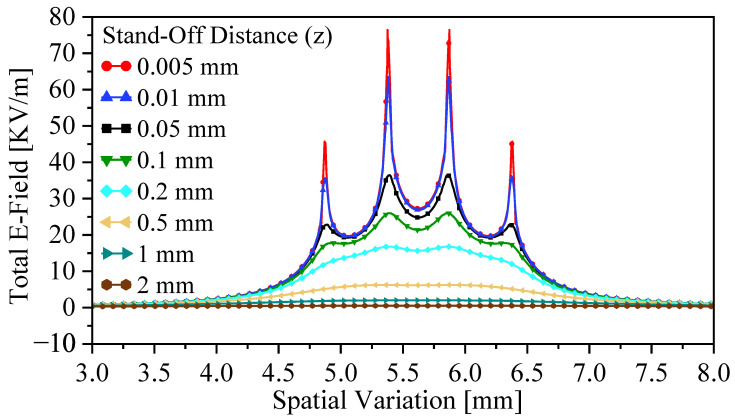
FSF as a function of the spatial variation and stand-off distance.

**Figure 12:**
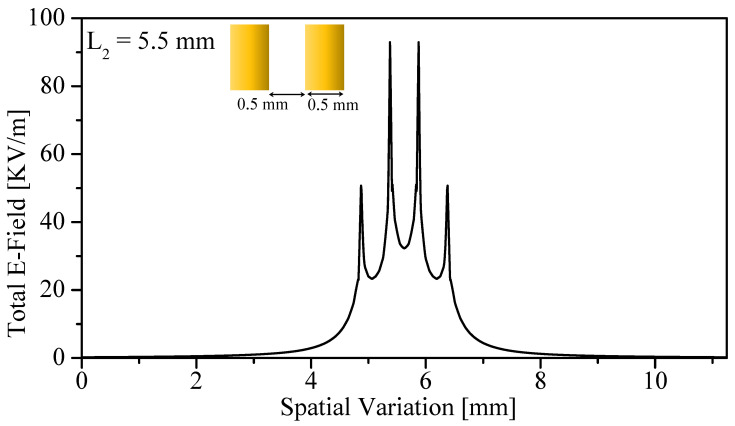
FSF as a function of the spatial variation, at $L_{2}$ = 5.5 mm, $b_{2}$ = 0.5 mm, and $b_{3}$ = 0.5 mm.

The field singularity behavior is further influenced by the stand-off distance *z*, as expected from Figure 4. Eight stand-off distances were investigated numerically (0.005–2 mm). As shown in Figure 11, FSF widens with increasing *z*, demonstrating that spatial resolution is directly linked to the stand-off distance. The effects of resonator scaling were also examined by reducing the resonator side length from 7.5 mm to 5.5 mm. Figure 12 reveals that the maximum field intensity increases by 19.87%, and the field-intensity difference between opposite sharp points grows from 33.12 to 42.2 kV/m. Thus, smaller resonators generate stronger singularity contrast, indicating that the sample–probe interaction is increasingly dominated by the sharp edges.

**Figure 13:**
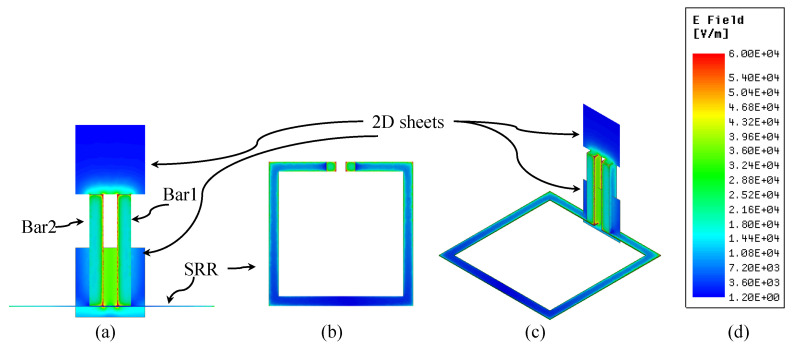
The total electric field of the proposed probe at two discontinuity positions at the bars’ tips and the transition region from the SRR’s plane to the two metal bars: (**a**) a side view near the bar tips (sensing region), (**b**) a top view of the SRR, (**c**) a perspective view, and (**d**) the field scaling.

To further illustrate the field behavior at geometrical discontinuities and to confirm the physical nature of the localized near field, Figure 13 presents representative two-dimensional electric-field distributions extracted from the full-wave simulation. The plots show a side view near the bar tips (sensing region), a top view of the SRR, and a three-dimensional perspective view, including the transition from the planar SRR-PCB region to the vertically extended metallic bars. These field distributions demonstrate continuous and physically consistent field localization at all critical discontinuities of the probe.

**Figure 14:**
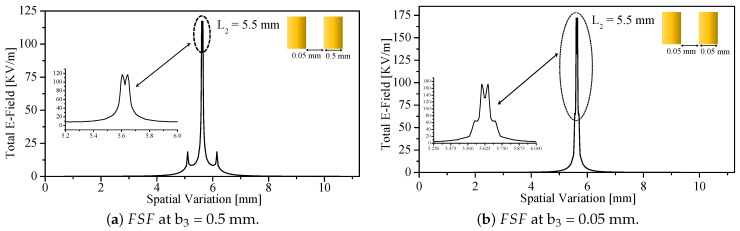
FSF as a function of the spatial variation, at $L_{2}$ = 5.5 mm and $b_{2}$ = 0.05 mm.

**Figure 15:**
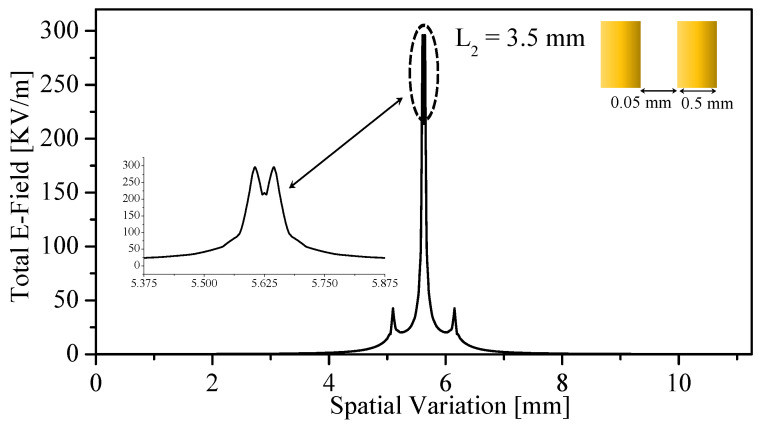
FSF as a function of the spatial variation, at $L_{2}$ = 3.5 mm, $b_{2}$ = 0.5 mm, and $b_{3}$ = 0.5 mm.

Finally, geometric variations of the split width $b_{2}$ and bar width $b_{3}$ were analyzed. Figure 14 demonstrates that reducing both $b_{2}$ and $b_{3}$ to 0.05 mm yields a more localized FSF with a peak magnitude of 172 kV/m. Similar behavior is observed for a smaller resonator with $L_{2}=3.5$ mm (Figure 15). These results confirm that FSF localization and singularity strength can be engineered by adjusting resonator and tip geometry.

### 3.2. One-Dimensional Object in One-Dimensional Image Space: Convolution with FSF

In near-field scanning microwave microscopy (NSMM), the resonance-frequency shift measured at each scan position corresponds to a spatially weighted interaction between the probe and the material beneath it. This weighting function is FSF, which directly leads to a one-dimensional convolution model. For an object with permittivity profile O(y), the resulting NSMM image is

(10) $$I\left(y\right)=\int_{-\infty }^{\infty }O\left(y-y^{\prime }\right)\;FSF\left(y^{\prime }\right)\;dy^{\prime }$$

Equation (10) reveals that the measured image is a blurred representation of the true object, where the degree of blurring is determined entirely by the spatial extent of FSF. A sharply localized FSF produces an image that closely matches the true object, whereas a wide FSF yields severe smearing and loss of detail. This model also explains how NSMM can surpass the Abbe diffraction limit: FSF contains high-spatial-frequency components, which contribute to the measurement when the stand-off distance is sufficiently small, as elaborated by (4).

To validate this model and quantify the influence of probe geometry on imaging performance, a one-dimensional numerical study was conducted using the previously extracted FSFs. A simple test object was defined: a homogeneous medium with $\epsilon_{r}=10$ containing a 4 mm wide central notch of $\epsilon_{r}=1$. This object emulates a localized defect within a high-permittivity slab. The profile O(y) is therefore piecewise-constant with a single low-permittivity region.

For each probe configuration, the one-dimensional FSF(y) was extracted from HFSS as the total electric-field magnitude along a line passing through the sensing region at a fixed stand-off distance of z=0.005 mm. Before performing the convolution, each FSF was shifted to a zero baseline and normalized such that

∫FSF(y)dy≈1

ensuring that the convolution behaves as a spatially weighted average rather than altering the overall image magnitude. For generating numerical results, the discrete version of (10) was implemented via

(11) $$I\left[n\right]=\sum_{k}O\left[k\right]\;FSF\left[n-k\right]\;\Delta y$$

**Figure 16:**
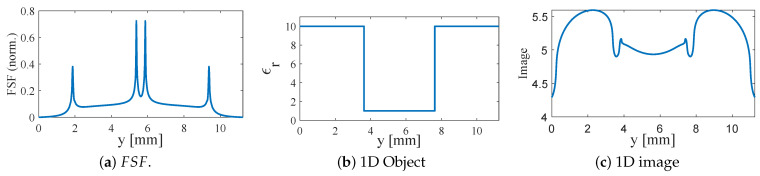
One-dimensional convolution results for the planar SRR without bars ($L_{2}=7.5$ mm, $b_{2}=0.5$ mm). (**a**) Normalized FSF extracted from the electric-field distribution at z=0.005 mm. (**b**) One-dimensional test object consisting of a 4 mm low-permittivity notch embedded in a high-permittivity background. (**c**) Resulting NSMM image obtained from the discrete convolution of the object with FSF, showing significant blurring due to the wide FSF.

**Figure 17:**
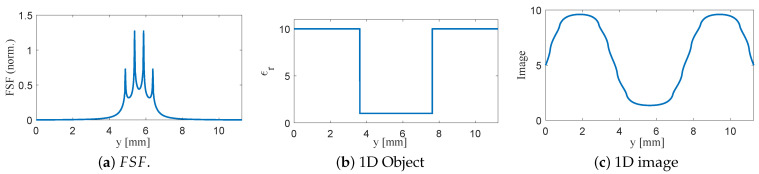
One-dimensional modeling using FSF of the SRR loaded with 0.5 mm vertical bars ($L_{2}=7.5$ mm, $b_{2}=0.5$ mm, $b_{3}=0.5$ mm). (**a**) Normalized FSF with increased field concentration near the sensing region. (**b**) The 1D dielectric test object consisting of a 4 mm low-permittivity notch within a high-permittivity background. (**c**) The resulting image obtained from the convolution I(y)=O(y)∗FSF(y), showing a deeper and narrower reconstructed notch compared to the planar SRR without bars.

**Figure 18:**
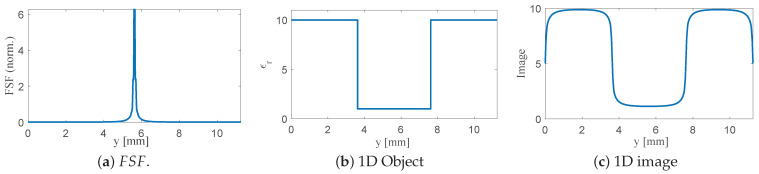
One-dimensional modeling using FSF of SRR loaded with thin 0.05 mm bars ($L_{2}=5.5$ mm, $b_{2}=0.05$ mm, $b_{3}=0.05$ mm). (**a**) Normalized FSF showing a narrow, dominant central lobe. (**b**) The 1D dielectric test object representing a 4 mm low-permittivity notch within a high-permittivity background. (**c**) The resulting image obtained from the convolution I(y)=O(y)∗FSF(y), the true profile with sharper transitions and reduced blurring compared to the thicker-bar case.

For each probe, three curves are plotted: the normalized FSF(y), the object O(y), and the resulting one-dimensional NSMM image I(y). Three probe configurations were investigated:

1. Planar SRR without bars ($L_{2}=7.5$ mm, $b_{2}=0.5$ mm). FSF exhibits four strong peaks associated with the 90° SRR edges and spreads across nearly the entire resonator width. Convolving this broad FSF with the 4 mm notch produces a wide, shallow dip; the notch appears much larger than its true size, and its boundaries are strongly blurred, as shown in Figure 16. The limiting factor is the wide FSF, not the size of the defect.

2. SRR with vertically extended bars ($L_{2}=7.5$ mm, $b_{2}=0.5$ mm, $b_{3}=0.5$ mm). The bars confine the electric field to the central region and suppress the contribution of the far edges. FSF becomes significantly more localized, resulting in a deeper and narrower reconstructed notch, as shown in Figure 17. Edge transitions are sharper than in case 1, confirming that 3D loading improves imaging resolution.

3. Reduced-length SRR with thin bars ($L_{2}=5.5$ mm, $b_{2}=0.05$ mm, $b_{3}=0.05$ mm). Reducing the resonator size and sharpening the bar geometry collapses FSF into a single dominant lobe with minimal side contributions. The convolved image closely follows the true 4 mm defect profile: the minimum is deeper, and the notch boundaries exhibit much steeper slopes, as shown in Figure 18. This configuration demonstrates a strong link between FSF localization and achievable resolution.

The progression from cases 1 to 3 demonstrates, in a controlled and quantitative manner, that engineering the probe geometry to tighten FSF directly enhances the spatial resolution of the NSMM system. These results also confirm the physical interpretation of the imaging process as a convolution and establish a foundation for the two-dimensional studies presented in the next subsection, where circular 2D defects embedded in a 2D dielectric slab are analyzed.

### 3.3. Two-Dimensional Object in Two-Dimensional Image Space: Convolution with 2D FSF

The one-dimensional convolution analysis in the previous subsection provides a clear demonstration of how the spatial extent of FSF dictates the attainable resolution in NSMM imaging. We now extend this analysis to two dimensions in order to model the imaging of circular 2D defects embedded in a 2D dielectric slab, which more closely reflects practical NSMM measurements.

A full two-dimensional FSF(x,y) can, in principle, be extracted directly from three-dimensional full-wave simulations by sampling the electric-field distribution over the entire plane at a fixed stand-off distance. However, because the sensing region of the proposed probe is symmetric with respect to the *x*- and *y*-axes, the electric-field distribution along the two directions is nearly identical. This symmetry allows the 2D FSF to be approximated as a separable function of the form

(12) $$FSF\left(x,y\right)\approx FSF_{1D}\left(x\right)\;FSF_{1D}\left(y\right),$$

where $FSF_{1D}\left(\right)$ is the normalized one-dimensional field profile extracted along either the *x*- or *y*-direction at z=0.005 mm. This separable approximation significantly reduces the computational cost and enables efficient 2D convolution without compromising the essential spatial characteristics of the field distribution. Using this model, a synthetic two-dimensional NSMM image is computed as

(13) $$I\left(x,y\right)=\;\int \int \;O\left(x-x^{\prime },\;y-y^{\prime }\right)\;FSF_{1D}\left(x^{\prime }\right)\;FSF_{1D}\left(y^{\prime }\right)\;dx^{\prime }\;dy^{\prime },$$

where O(x,y) denotes the 2D dielectric object. In this work, O(x,y) represents a high-permittivity slab ($\epsilon_{r}=10$) containing a centrally located circular void of diameter 4 mm with $\epsilon_{r}=1$. The domain is discretized to match the sampling of the 1D FSF, and the convolution in (13) is implemented using a two-dimensional discrete convolution with the separable approximation of FSF, $FSF_{1D}\left(x\right)\;FSF_{1D}\left(y\right)$.

**Figure 19:**
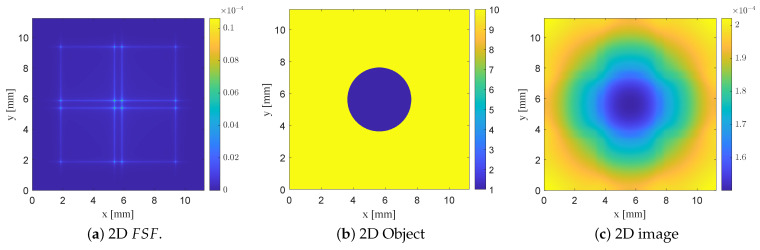
Two-dimensional convolution modeling using FSF of the planar SRR probe without vertically extended bars ($L_{2}=7.5\;\mathrm{mm},b_{2}=0.5\;\mathrm{mm}$). (**a**) Normalized separable 2D FSF(x,y), obtained from the 1D field profile. (**b**) Two-dimensional dielectric test object representing a 4 mm circular low-permittivity inclusion $\left(\epsilon_{r}=1\right)$ embedded in a high-permittivity background $\left(\epsilon_{r}=10\right)$. (**c**) Resulting NSMM image I(x,y)=O(x,y)∗FSF(x,y), in which the defect appears enlarged with diffuse edges due to the broad FSF of the unloaded SRR probe.

For each probe configuration examined previously (the planar SRR and SRR loaded with bars), a corresponding two-dimensional image was generated using the separable 2D FSF(x,y). The resulting images clearly illustrate how the degree of field localization governs the quality of the reconstructed dielectric inclusion. In particular, Figure 19 illustrates two-dimensional convolution modeling using FSF of the planar SRR probe without vertically extended bars $\left(L_{2}=7.5\;\mathrm{mm},\;b_{2}=0.5\;\mathrm{mm}\right)$. The normalized separable 2D FSF(x,y) is reconstructed from the corresponding 1D field profile, and a 2D dielectric test object consisting of a 4 mm circular low-permittivity inclusion ($\varepsilon_{r}=1$) embedded in a high-permittivity background ($\varepsilon_{r}=10$) is used. The resulting NSMM image, computed using (13), demonstrates that the defect appears enlarged with diffuse and poorly defined edges due to the broad FSF of the unloaded planar SRR probe.

**Figure 20:**
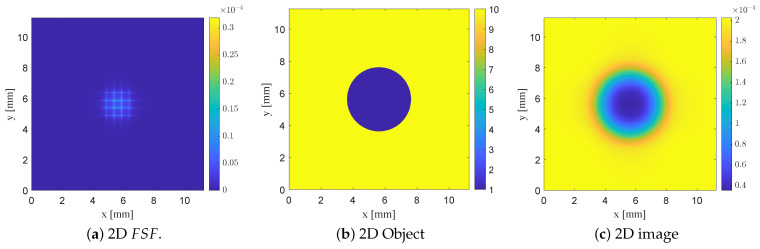
Two-dimensional convolution modeling using FSF of the SRR probe loaded with vertically extended metallic bars ($L_{2}=7.5\;\mathrm{mm},b_{2}=0.5\;\mathrm{mm},b_{3}=0.5\;\mathrm{mm}$). (**a**) Normalized separable 2D field-spread function FSF(x,y) generated from the extracted 1D field profile. (**b**) Two-dimensional dielectric test object containing a 4 mm circular low-permittivity inclusion $\left(\epsilon_{r}=1\right)$ within a high-permittivity background $\left(\epsilon_{r}=\right.$ 10). (**c**) Resulting NSMM image I(x,y)=O(x,y)∗FSF(x,y) showing a more confined response compared to the planar SRR.

**Figure 21:**
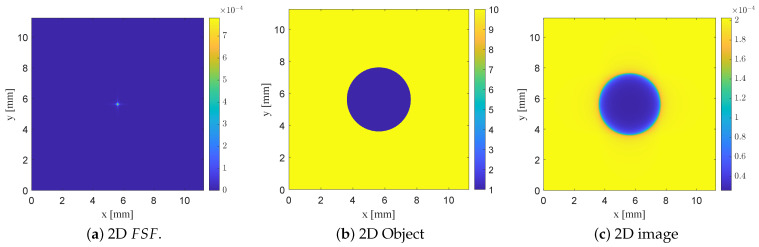
Two-dimensional convolution modeling using the FSF of the SRR probe loaded with thin vertically extended bars ($L_{2}=5.5\;\mathrm{mm},b_{2}=0.05\;\mathrm{mm},b_{3}=0.05\;\mathrm{mm}$). (**a**) Normalized separable 2D FSF(x,y) derived from the localized 1D field profile. (**b**) Two-dimensional dielectric test object with a 4 mm circular low-permittivity inclusion $\left(\epsilon_{r}=1\right)$ embedded in a high-permittivity background $\left(\epsilon_{r}=10\right)$. (**c**) Resulting NSMM image I(x,y)=O(x,y)∗FSF(x,y), showing improved confinement of the reconstructed defect consistent with the more concentrated FSF produced by the thin-bar geometry.

Introducing vertically extended bars narrows and localizes FSF, thereby improving spatial confinement and yielding a reconstructed defect that more closely preserves the circular geometry of the object, with sharper transitions at its edges, as shown in Figure 20. The most localized FSF, obtained using the thin-bar configuration $\left(L_{2}=5.5\;\mathrm{mm},\;b_{2}=b_{3}=0.5\;\mathrm{mm}\right)$, produces the highest-fidelity reconstruction, with the imaged void closely approaching the true size and shape of the underlying circular defect as shown in Figure 21. These 2D convolution results parallel the trends observed in the 1D analysis, showing that increasingly localized FSF profiles systematically enhance the fidelity of the forward-modeled NSMM image. Taken together, the 1D and 2D studies confirm the predictive capability of the FSF-based modeling framework and quantitatively demonstrate how probe geometry directly governs the achievable resolution in near-field scanning microwave microscopy.

### 3.4. k-Space Analysis: Spectrum Content

The 1D convolution model in (10) admits a simple interpretation in the spatial-frequency (or *k*-space) domain. Denoting the Fourier transform of a function f(y) by F{f}(k), the convolution theorem gives

(14) $$I\left(y\right)=O\left(y\right)*FSF\left(y\right)\;⟺\;\tilde{I}\left(k\right)=\tilde{O}\left(k\right)\;\tilde{FSF}\left(k\right)$$

where $\tilde{O}\left(k\right)$, $\tilde{FSF}\left(k\right)$, and $\tilde{I}\left(k\right)$ are the spectra of the object, the field-spread function, and the resulting image, respectively. Thus, in *k*-space, the NSMM probe behaves as a spatial filter whose transfer function is precisely $\tilde{FSF}\left(k\right)$.

For the 1D test object used in the previous subsection (a high-permittivity background $\epsilon_{r}=10$ with a 4 mm notch of $\epsilon_{r}=1$), the spectrum $\tilde{O}\left(k\right)$ is fixed for all probe configurations. Numerically, $\tilde{O}\left(k\right)$ was obtained from the discrete spatial samples using an FFT with appropriate scaling and a frequency axis

(15) $$k=\frac{2\pi }{N\;\Delta y}\;n,\;n=-\frac{N}{2},\ldots ,\frac{N}{2}-1$$

where Δy is the spatial step and *N* is the number of samples. As expected for a piecewise-constant profile with two sharp edges, $|\tilde{O}\left(k\right)|$ exhibits a dominant low-*k* peak around k=0 with slowly decaying sidelobes.

**Figure 22:**
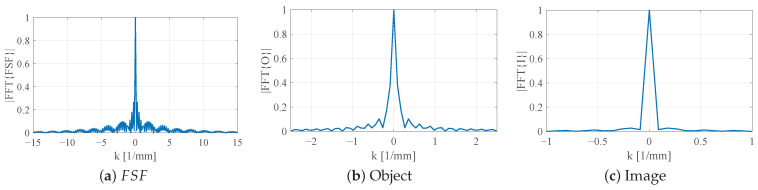
Fourier-domain characterization of the planar SRR probe without vertically extended bars ($L_{2}=7.5\mathrm{mm},b_{2}=0.5\;\mathrm{mm}$). (**a**) Magnitude of the 1D FSF spectrum |FFT{FSF}|, showing a narrow central peak and rapidly decaying high-*k* components. (**b**) Spectrum of the 4 mm notch object |FFT{O}|. (**c**) Spectrum of the resulting image |FFT{I}|, explaining the pronounced blurring observed in the spatial-domain image.

Figure 22 illustrates the *k*-space behavior for the planar SRR probe without bars ($L_{2}=7.5$ mm, $b_{2}=0.5$ mm). The magnitude of FSF spectrum, $|\tilde{FSF}\left(k\right)|$, is strongly concentrated near k=0 with relatively narrow bandwidth and modest sidelobes. Consequently, the product $|\tilde{I}\left(k\right)|=|\tilde{O}\left(k\right)\;\tilde{FSF}\left(k\right)|$ remains dominated by low spatial frequencies, where most of the higher-*k* components of the object are strongly attenuated. In the spatial domain, this manifests as a broadened, shallow dip in I(y) and poor localization of the 4 mm notch.

**Figure 23:**
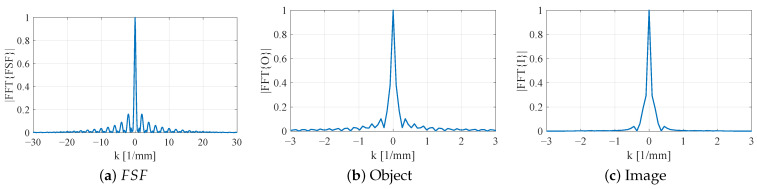
Fourier-domain characterization of the SRR probe loaded with vertically extended metallic bars ($L_{2}=7.5\;\mathrm{mm},b_{2}=b_{3}=0.5\;\mathrm{mm}$). (**a**) FSF spectrum |FFT{FSF}|, showing increased high-*k*. (**b**) Spectrum of the 4 mm notch object |FFT{O}|. (**c**) Spectrum of the resulting image |FFT{I}|, where multiplication by the less band-limited FSF preserves more of the object’s high-*k* components.

When the SRR is loaded with vertically extended metallic bars while keeping $L_{2}=7.5$ mm and $b_{2}=b_{3}=0.5$ mm, FSF becomes more localized in space, and its spectrum $|\tilde{FSF}\left(k\right)|$ correspondingly broadens, as shown in Figure 23. The central lobe in *k*-space is wider, and the sidelobes extend to larger |k| compared to the planar case. The resulting image spectrum $|\tilde{I}\left(k\right)|$ therefore retains a larger portion of the object’s mid-range spatial frequencies. This increase in effective bandwidth explains the deeper and more confined notch observed in the corresponding 1D image.

**Figure 24:**
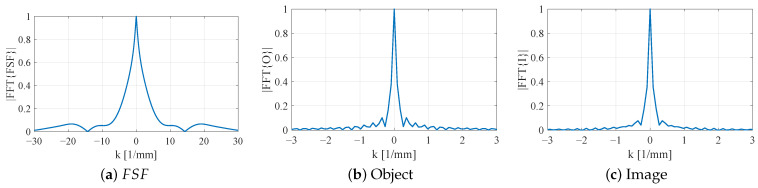
One-dimensional forward-modeling using FSF of the SRR loaded with thin 0.05 mm bars $\left(L_{2}=5.5\;\mathrm{mm},\;b_{2}=0.05\;\mathrm{mm},\;b_{3}=0.05\;\mathrm{mm}\right)$. (**a**) Normalized FSF showing a narrow, dominant central lobe. (**b**) The 1D dielectric test object representing a 4 mm low-permittivity notch within a high-permittivity background. (**c**) The resulting image showing that the reconstructed notch closely follows the true profile with sharper transitions and reduced blurring.

A further reduction in the resonator side length to $L_{2}=5.5$ mm with thin bars ($b_{2}=b_{3}=0.05$ mm) produces an even tighter FSF in the spatial domain and, by the Fourier uncertainty principle, a substantially broader $|\tilde{FSF}\left(k\right)|$ (Figure 24a). In this configuration, significant spectral content persists out to much higher |k| values. The product $|\tilde{I}\left(k\right)|$ now closely follows $|\tilde{O}\left(k\right)|$ over a much wider *k*-range, indicating that the probe transmits most of the object’s high-*k* information. In real space, this corresponds to an image I(y) that nearly reproduces the true 4 mm notch width with sharply defined transitions.

Overall, the *k*-space analysis confirms the qualitative trends observed in the spatial-domain convolution results. As FSF is progressively localized through 3D loading and resonator miniaturization, its spectrum broadens and admits higher spatial frequencies. This enhanced high-*k* content directly correlates with improved resolution and more faithful reconstruction of subwavelength features in NSMM imaging.

## 4. Case Studies: Experimental Validation

### 4.1. A Dielectric Slab with a Cylindrical-Void Defect

**Figure 25:**
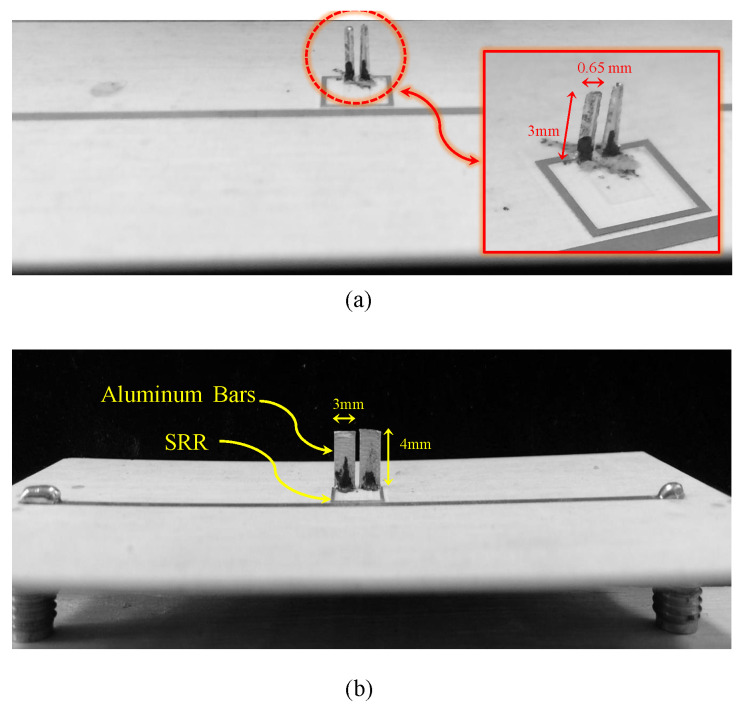
Fabricated SRR-based near-field sensors with vertically extended metallic probes. (**a**) An SRR with L=7.5 mm, bar length $L_{\mathrm{sp}}=3$ mm, and bar widths $b_{2}=0.5$ mm, $b_{3}=0.65$ mm, and $b_{1}=0.25$ for the probe and 0.5 mm for SRR. (**b**) L=7.5 mm, bar length $L_{\mathrm{sp}}=4$ mm, and bar widths $b_{2}=0.25$ mm, $b_{3}=3$ mm, and $b_{1}=0.25$ for the probe and 0.5 mm for SRR.

**Figure 26:**
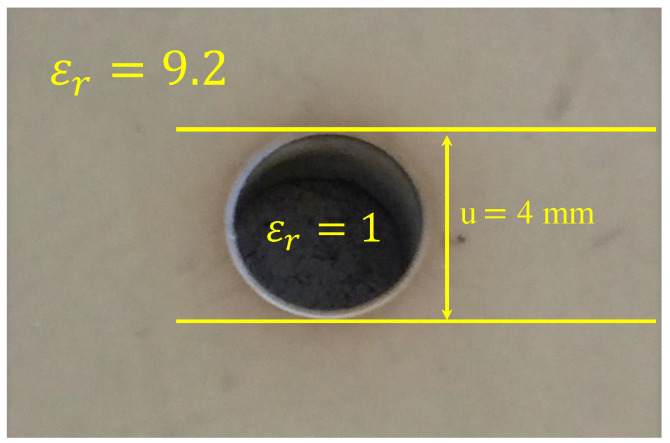
Fabricated dielectric slab ($\varepsilon_{r}=9.2$) containing a cylindrical air-filled void ($\varepsilon_{r}=1$) with a diameter of u=4mm.

**Figure 27:**
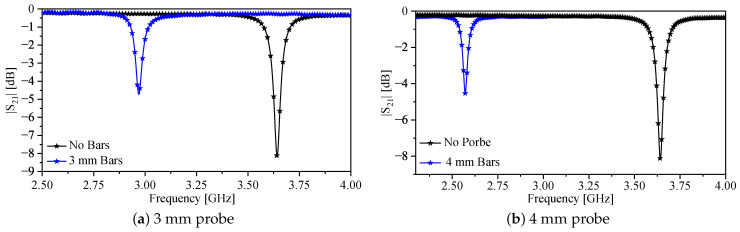
Transmission coefficient $|S_{21}|$ for SRR probes with (blue) and without (black) metallic bars: (**a**) 3 mm probe; (**b**) 4 mm probe.

To experimentally validate the proposed NSMM technique and to demonstrate the imaging capability of two-fabricated probes, a dielectric slab containing a cylindrical void was scanned using the SRR-bar sensors. The first fabricated probe consisted of an SRR with L=7.5 mm, bar length $L_{\mathrm{sp}}=3$ mm, and bar widths $b_{2}=0.5$ mm, $b_{3}=0.65$ mm, and $b_{1}=0.25$ for the probe and 0.5 mm for SRR, whereas the second probe has the following specifications: L=7.5 mm, bar length $L_{\mathrm{sp}}=4$ mm, and bar widths $b_{2}=0.25$ mm, $b_{3}=3$ mm, and $b_{1}=0.25$ for the probe and 0.5 mm for SRR. Both probes are shown in Figure 25. The material under test (MUT) was a ceramic slab with a height of 6 mm, a length of approximately 105 mm, a width of approximately 60 mm, and a relative permittivity $\varepsilon_{r}=9.2$. A cylindrical air-filled cavity of diameter u=4 mm was drilled through the slab, as shown in Figure 26. The probes were excited using a vector network analyzer (VNA), and the transmission response $|S_{21}|$ was recorded. Figure 27 shows the response of the 3 mm probes (a) and 4 mm probes (b) in free space, respectively, which serves as the reference for all subsequent measurements.

**Figure 28:**
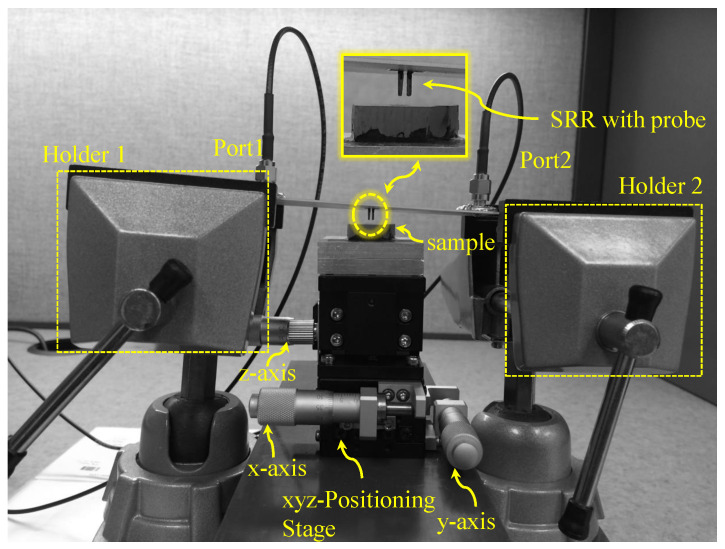
Experimental setup for the two-dimensional near-field scan. The SRR probe is fixed in a position using two holders and an XYZ manual positioning stage underneath the sample, with microwave ports (Port 1 and Port 2) connected to the sensor to the VNA. A stand-off distance of 5 μm was set after calibration, and the sample was scanned over a 28×32 grid with a 0.25 mm step in both *x*- and *y*-directions.

**Figure 29:**
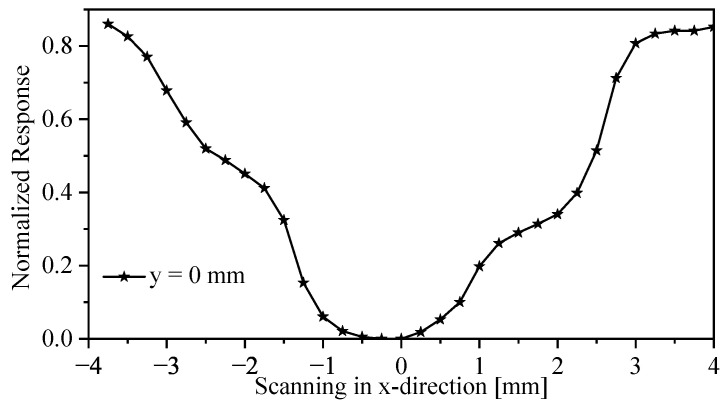
Normalized response of line profile through the center, where a horizontal cut at the center row in the original image was taken.

For the two-dimensional scan, the SRR probe is fixed in a position using two holders and an XYZ manual positioning stage underneath the sample, with microwave ports (Port 1 and Port 2) connected to the sensor to the VNA, shown in Figure 28. The system was first calibrated by gently touching the probe to the slab surface, and then the stand-off distance was set to 5 μm to ensure maximum sensitivity. The MUT was scanned over a 28×32 grid with a spatial step of 0.25 mm in both *x*- and *y*-directions. The maximum observed resonance-frequency shift ($\Delta f_{max}$) corresponds to the region where the probe directly senses the high-permittivity dielectric ($\varepsilon_{r}=9.2$). This maximum value was used to normalize the measured frequency shifts for all scan points (e.g., the reference point is $\Delta f_{max}-\Delta f_{max}=0$). Figure 29 shows the normalized resonance-frequency shift versus the scanning in the x-direction, where the y is fixed at 0 mm. The mild asymmetry observed in the line-scan can be attributed to fabrication tolerances in the planar SRR and the bars’ tips ($b_{1}$ and $b_{2}$).

The relationship between the probe field distribution and the measured image is confirmed by comparing the modeled results in Figure 18c and Figure 21c with the experimental scan shown in Figure 29. For instance, in Figure 17c and Figure 18c, the reconstructed one-dimensional images were obtained by convolving the dielectric object with the corresponding FSF of the SRR probe. The resulting notches exhibit the expected broadening and smoothing imposed by the probe’s field distribution, consistent with the linear imaging model I(y)=O(y)∗FSF(y). The experimental profile in Figure 29 reproduces the same characteristic shape when scanning across the physical dielectric notch. The close agreement between the measured response and the predicted convolved profiles provides strong validation that the imaging mechanism of the proposed probe is governed by the convolution between the object and the near-field distribution of the probe. This confirms that the probe does not directly map the object itself, but rather maps the object after being filtered through its spatial field sensitivity.

**Figure 30:**
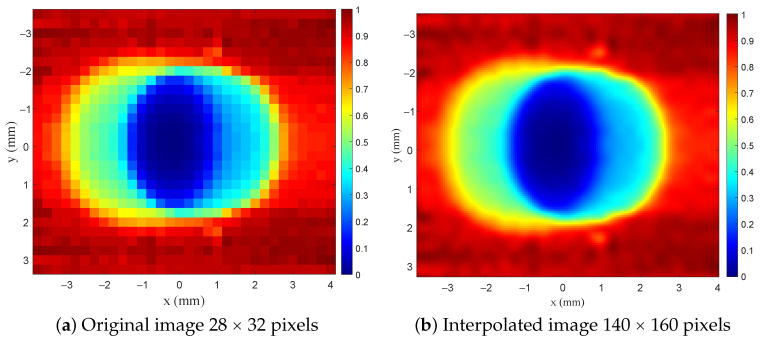
Experimental results of a 2D image of the slab with the cylindrical void of 4 mm: an original and an interpolated one.

Furthermore, Figure 30a,b show the reconstructed two-dimensional images of the void using the raw and interpolated data, respectively. The cubic-spline interpolation is used solely for visualization to smooth the edges; the raw data are used for all quantitative assessments. Although the void is circular, the reconstructed image appears elliptical, with a wider spread along the *x*-direction. This is not an artifact but can be attributed to a physical consequence of the *anisotropic FSF* of the fabricated probe. The SRR–bar geometry produces a strongly confined field in the *y*-direction (between the bar tips) and a wider fringing-field distribution along the *x*-direction (parallel to the microstrip excitation). Therefore, the convolution of the circular void with the anisotropic FSF yields an elliptical signature in the measured image. This experimentally validates the FSF-based imaging model introduced earlier in the paper.

### 4.2. Surface Detection of Different Materials

**Figure 31:**
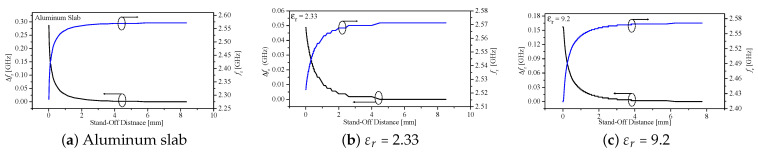
Experimental results: resonance frequency and resonance-frequency shift versus the stand-off distance, varied from 5 μm (reference position) to approximately 8.35 mm. The blue line is for the resonance frequency, whereas the black line is for the resonance frequency shift.

To further examine the sensing capability of the proposed technique, a second probe (4 mm probe) was utilized for surface detection of different materials. Three materials were tested: an aluminum slab, a dielectric slab with $\varepsilon_{r}=2.33$, and a dielectric slab with $\varepsilon_{r}=9.2$. For each material, the resonance frequency and the corresponding shift were recorded while the stand-off distance was varied from 5 μm (reference position) to approximately 8.35 mm. The results for the aluminum slab are shown in Figure 31a. The dielectric materials with $\varepsilon_{r}=2.33$ and $\varepsilon_{r}=9.2$ are shown in Figure 31b and Figure 31c, respectively. The monotonic decrease in the frequency shift with increasing stand-off distance reflects the rapid attenuation of the high-spatial-frequency components (k-space spectrum) of the probe’s near-field. Moreover, the distinct resonance trajectories measured for the three materials demonstrate the probe’s ability to discriminate surfaces based on their effective materials.

## 5. Conclusions

This work proposed an NSMM probe based on a split-ring resonator (SRR) loaded with vertically extended metallic bars. The metallic bars enhance the sensing area, where they produce strong electric-field localization at the sharp tips. In addition, the sensing area becomes farther away from TL, avoiding any parasitic coupling. The proposed probe was investigated numerically using a 3D full-wave simulation. It shows that at the sharp tips (90° conducting edges), the electric-field profile is enhanced dramatically and becomes localized, which significantly enhances sensitivity. To quantify the localization of the concentrated field profile in space, a function named FSF, similar to the point-spread function in optics, was introduced. Using the numerical simulation, FSF was extracted and analyzed for multiple probe tips’ dimensions. We found that reducing resonator size, split width, and bar width produced sharper and more localized FSFs with higher peaks. Furthermore, a corresponding *k*-space analysis demonstrated that localized FSFs contain broader spatial-frequency content, associated with higher-*k* components and subwavelength features, if used for imaging. Furthermore, the 1D and 2D convolution techniques were introduced to convolve the FSFs with the object’s profile to produce images. The numerical studies showed that broader FSFs produce strongly blurred images, while highly localized FSFs produce enhanced images of dielectric defects. The probes were fabricated using PCB technology with aluminum bars that were attached using a conductive adhesive. In addition, a sample of a dielectric slab was prepared for the test, where the slab has a cylindrical void. The experimental image of the dielectric slab showed strong agreement with the convolution-based predictions. Therefore, the proposed technique provides a compact probe with low radiation and a high sensitivity and resolution for NSMM. The combined FSF and *k*-space concept in the analysis of the proposed probe provides a methodology and guidance for the design of future NSMM near-field probes. 

## Figures and Tables

**Table 1:** Design specification.

Sensor Type	$L_{2}$ (mm)	$W_{\mathrm{TL}}$ (mm)	$b_{1}$ (mm)	$b_{2}$ (mm)	b (mm)	$W_{\mathrm{sub}}$ (mm)	$L_{\mathrm{SP}}$ (mm)	$L_{G}$ (mm)	$W_{G}$ (mm)	$W_{\mathrm{SP}}$ (mm)
**SRR (No Bars)**	7.5	1.63	0.5	0.5	0.5	0.76	NA	100	50	0.5 mm
**SRR (with Bars)**	∼	∼	Vari.	Vari.	∼	∼	Vari.	∼	∼	∼

## Data Availability

Data generated during the study are contained within the article.

## Abbreviations

The following abbreviations are used in this manuscript:

MUT	Material under test
TL	Transmission line
SRR	Split-ring resonator
CSRR	Complementary split-ring resonator
SUT	Sample under test
FSF	Field-spread function
FWHM	Full width at half maximum
